# The effect of aging on the fracture resistance of different types of screw-cement-retained implant-supported zirconia-based restorations

**DOI:** 10.1371/journal.pone.0270527

**Published:** 2022-06-24

**Authors:** Safoora Sahebi, Rashin Giti, Arian Sherafati

**Affiliations:** 1 Department of Endodontics, School of Dentistry, Shiraz University of Medical Sciences, Shiraz, Fars, Iran; 2 Department of Prosthodontics, School of Dentistry, Shiraz University of Medical Sciences, Shiraz, Fars, Iran; 3 Student Research Committee, School of Dentistry, Shiraz University of Medical Sciences, Shiraz, Fars, Iran; Semnan University, ISLAMIC REPUBLIC OF IRAN

## Abstract

Structural durability of screw-cement-retained implant-supported zirconia-based restorations is an important factor in choosing the best type of restoration for clinical use. This study aimed to evaluate the effects of thermocycling on the fracture resistance of different types of screw-cement-retained implant-supported zirconia-based restoration. Two experimental groups (monolithic zirconia and porcelain-veneered zirconia) and a control group of porcelain-fused-to-metal restorations were fabricated via CAD-CAM (*n* = 14 per group). Half of the specimens of each group (*n* = 7) were subjected to 10000 thermal cycles. The compressive force was applied and the force leading to fracture was measured by using a Universal Testing Machine. The fractured modes were classified under a scanning electron microscope. The data were analyzed through two-way ANOVA, one-way ANOVA, and independent samples t-test (*α* = 0.05). Among the non-thermocycled subgroups, the monolithic zirconia specimens were significantly more fracture-resistant than the porcelain-veneered zirconia and porcelain-fused-to-metal groups (*P*<0.05); but it was not the same with aging (*P*>0.05). Thermocycling decreased the fracture resistance of all groups; however, the difference was not statistically significant (*P*<0.05). The monolithic zirconia presented higher fracture resistance than the bilayered restorations for screw-cement retained implant-supported restorations. Thermocycling decreased the fracture resistance of all types of restorations insignificantly which can be clinically important.

## 1. Introduction

The durability of screw-cemented implant-supported restorations is critical for the long-term success of these restorations [1]. Implant-supported restorations can be fixed by cement- or screw-retaining restorations on the implant abutments. Cemented restorations do not interfere with occlusion or esthetics as they do not require an access hole; however, their removal and cleaning can be challenging. Screw-retained restorations are esthetically poor and probable of screw loosening, but they are retrievable and have better marginal fit [2]. To benefit from the advantages of both types, cement-screw-retained restorations or “combination implant crowns” were introduced by McGlumphy in 1992 [3, 4].

Zirconium dioxide (zirconia) based restorations have superb biocompatibility and favorable mechanical properties and are widely used in restorative materials, implant abutments, and frameworks for implant-supported restorations [1, 5]. Although zirconia restorations have shown promising survival rates, fracture of veneering porcelain occurs frequently [6–8]. Monolithic zirconia restorations were found to be excellent to prevent veneering porcelain fracture in the short-term clinical evaluations [9, 10].

Hussein et al. [11] reported that the preparation of the screw access channel did not affect the fatigue loads of zirconia crowns. Malpartida-Carrillo et al. [12] reported that cement and combined cement- and screw-retained metal-ceramic molar restorations had comparable fracture resistance with 15°-angulated abutments. On the contrary, Mallmann et al. [13] found that screw-retained zirconia-based implant-supported fixed dental prostheses were less fracture-resistant than cement-retained prostheses. They also concluded that zirconia-based restorations were more fracture-resistant than metal-based restorations. Moreover, another study showed that preparing a screw access hole in cement-retained implant-supported zirconia-based crown resulted in decreased fracture resistance of the restoration and adding a ledge in the zirconia framework around the access hole might increase the fracture resistance of the restoration [14]. It has also been reported that the type of sealant materials influenced the fracture resistance of resin composite applied in sealing screw access hole in screwed implants [15]. In two different studies, Honda et al. [1, 2] detected that monolithic screw-retained implant-supported and tooth-supported restorations were significantly more fracture-resistant than bilayered restorations. Likewise, Johansson et al. [16] showed that the fracture strength of monolithic high-translucent zirconia restorations was higher than porcelain-veneered crowns and lithium disilicate crowns for non-implant restorations. A different study found that after thermo-mechanical aging, the fracture strength of aged monolithic zirconia was more than aged bilayer zirconia-based crowns [17]. Al-Zordk et al. [18] detected that the fracture resistance of implant-supported restorations based on zirconia was more than lithium disilicate and ceramic-reinforced polyetheretherketone restorations after thermal aging.

Although the mentioned studies evaluated the effect of different types of restoration on the fracture resistance of both tooth- and implant-supported restorations either with or without thermal aging, no study has evaluated the simultaneous effect of thermal aging and type of restoration on the fracture resistance of screw-cement-retained implant-supported zirconia-based restorations. Consequently, the present study was designed to evaluate the effect of thermocycling on the fracture resistance of screw-cemented implant-supported monolithic zirconia and porcelain veneered zirconia restorations.

The null hypotheses were that the three types of restoration would not have different fracture resistance regardless of thermocycling and that thermocycling would not affect the fracture resistance of different screw-cement-retained implant-supported zirconia-based restorations.

## 2. Materials and methods

### 2.1. Preparation of specimens

**Fig 1:**
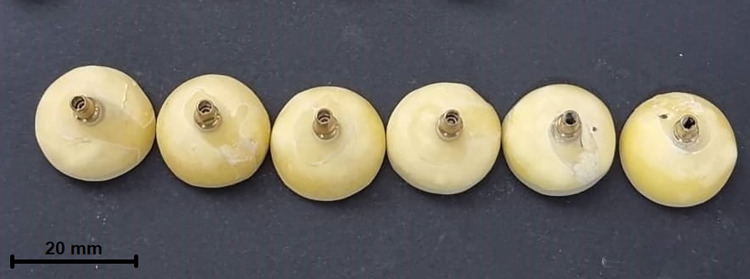
Titanium-based abutments and analogs embedded in acrylic material.

Six implant analogs (5×11.5 mm [diameter×height]) (Dio Implant, South Korea) were used to replicate missing maxillary first premolar teeth. The analogs were embedded and fixed in acrylic (Acropars, Iran), and also filled with acrylic up to 1 mm beneath the connection. Six titanium-based abutments (5-mm platform diameter, 7.5-mm width, 7-mm height) were screwed to the analogs by using a hand-driven wrench (Healteckorea, South Korea) up to 30 N (**Fig 1**). Forty-two implant-supported crowns were assigned to 3 groups (n = 14 per group) of porcelain-veneered zirconia (PVZ), monolithic zirconia (MZ), and porcelain-fused-to-metal (PFM) serving as the control group.

#### 2.1.1. Porcelain-veneered zirconia group

**Fig 2:**
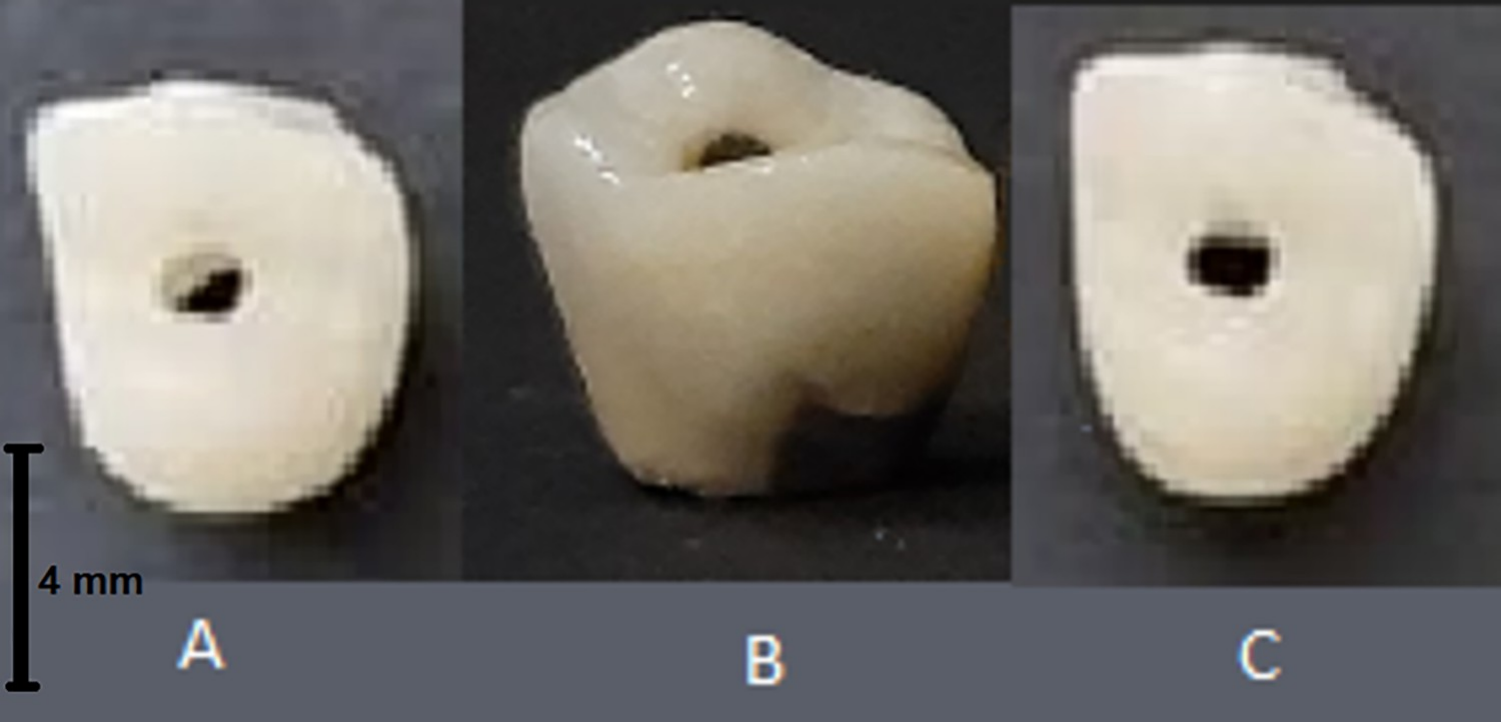
The study groups. a: porcelain-veneered zirconia, b: porcelain-fused-to-metal, c: monolithic zirconia.

Fourteen zirconia frames (Dental Direkt, Germany) were manufactured by using computer-aided design/computer-aided manufacturing ([CAD/CAM], imes-icore 350i, Germany). Screw access holes (2.7 mm) were created on the occlusal surfaces. The frameworks were then airborne particle-abraded with 50-μm aluminum oxide for 20 seconds at 0.2 MPa, as described by previous studies [1, 2]. Finally, feldspathic porcelain (Creation, Austria) was veneered onto the zirconia frameworks according to the manufacturer’s instructions (**Fig 2A**).

#### 2.1.2. Porcelain-fused-to-metal group

To create the metal frameworks by using the CAM machine, wax patterns were fabricated on the titanium-base abutments and were cast with non-precious metal alloy (Scheftner, Germany). Feldspathic porcelain (IPS Inline, Ivoclar Vivadent, USA) was then fired onto the surface of the metal frameworks to create the same contour as the two other groups (**Fig 2B**).

#### 2.1.3. Monolithic zirconia group

Monolithic zirconia crown patterns were waxed up on titanium abutments by using light-curing resin (Photec; China) with the same specifications as the PVZ group. Wax patterns and abutments were then scanned with the CAD software (FastDesign, Glidewell, United States) and milled with the CAM machine from monolithic zirconia blocks (Dental Direkt, Germany) (**Fig 2C**).

Wax plugs were used to preserve the screws from cement so they can be later accessed and tightened. The restorations were cemented onto the abutments by using a dual-cure resin cement (Panavia F2.0, Kuraray, Japan). The abutment channels and the screw access holes were filled with resin composite (Estelite, Tokuyama, Japan) and were left to set completely for 24 hours.

### 2.2. Thermocycling and compressive force test

**Fig 3:**
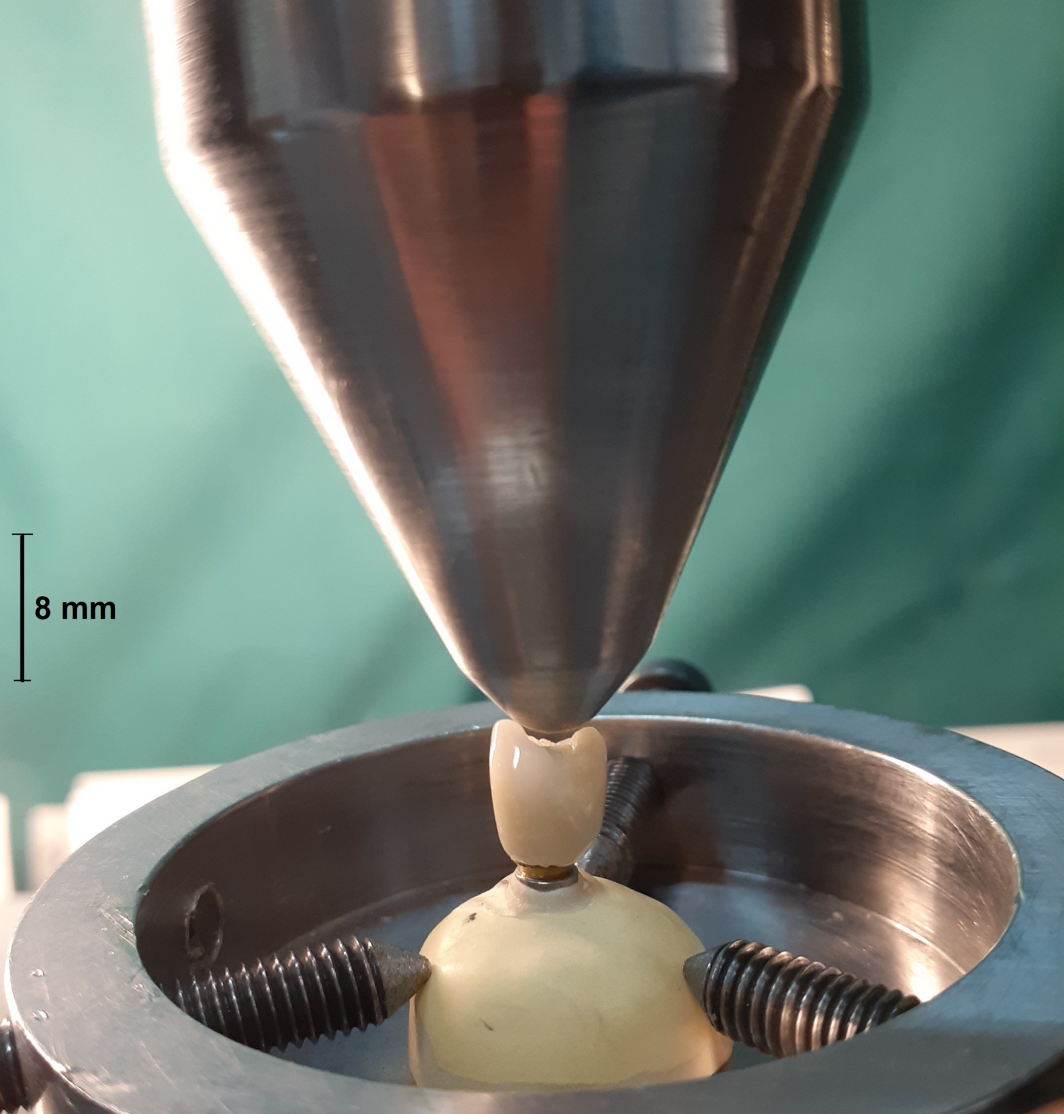
A specimen being tested in the universal testing machine.

**Fig 4:**
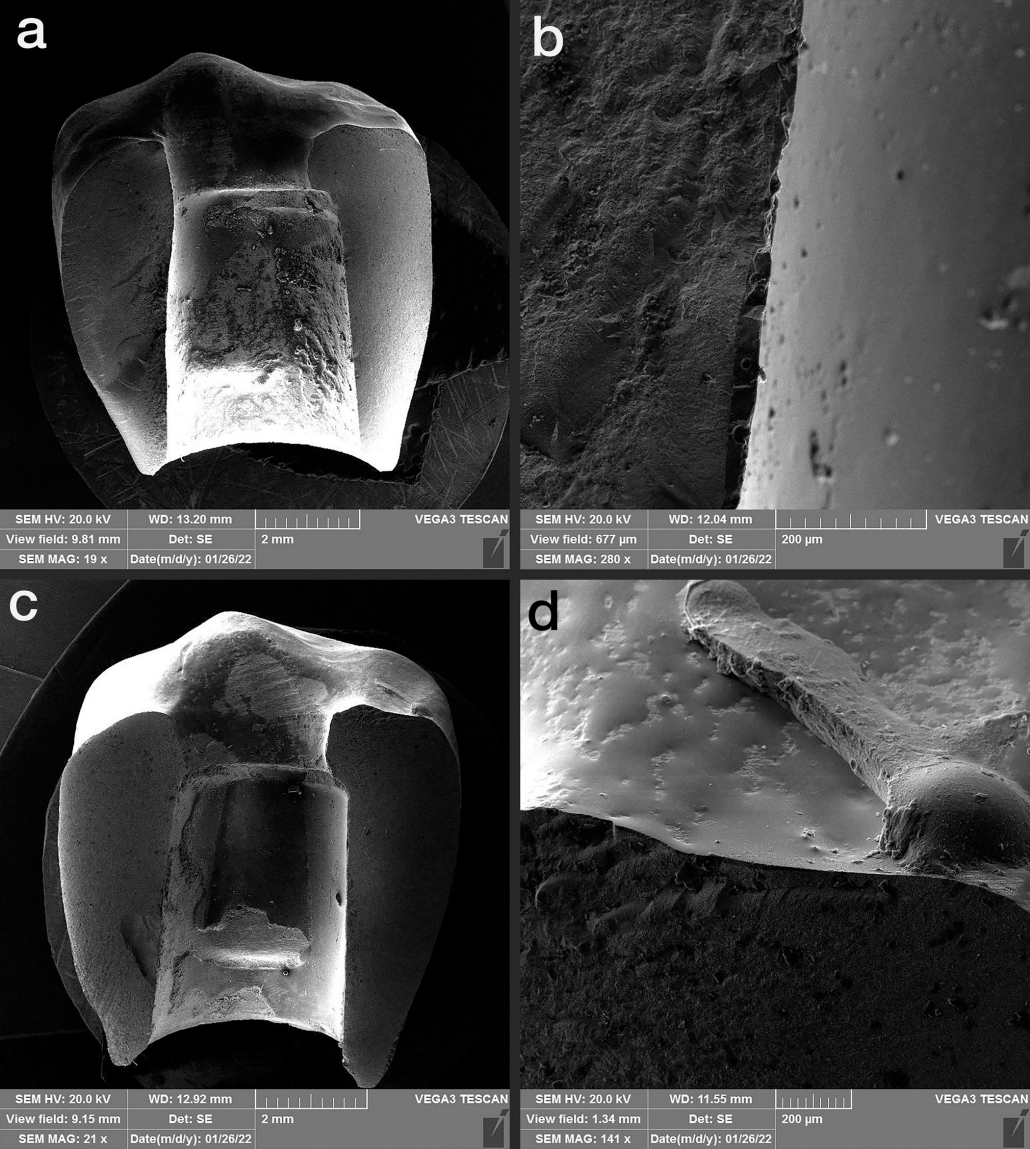
SEM images of monolithic zirconia. a and b: thermocycled, c and d: non-thermocycled.

**Fig 5:**
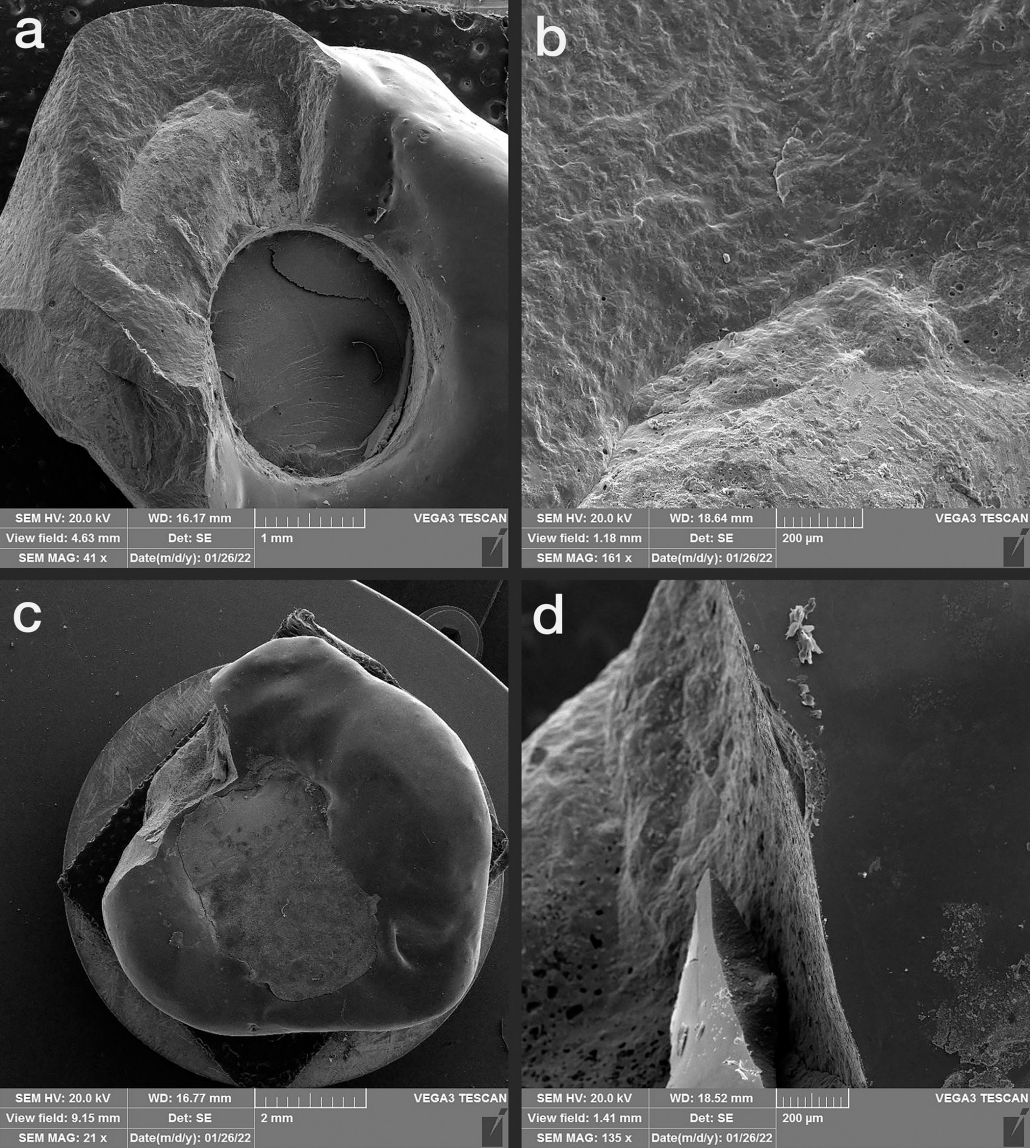
SEM images of the porcelain-fused-to-metal specimen. a and b: thermocycled, c and d: non-thermocycled).

**Fig 6:**
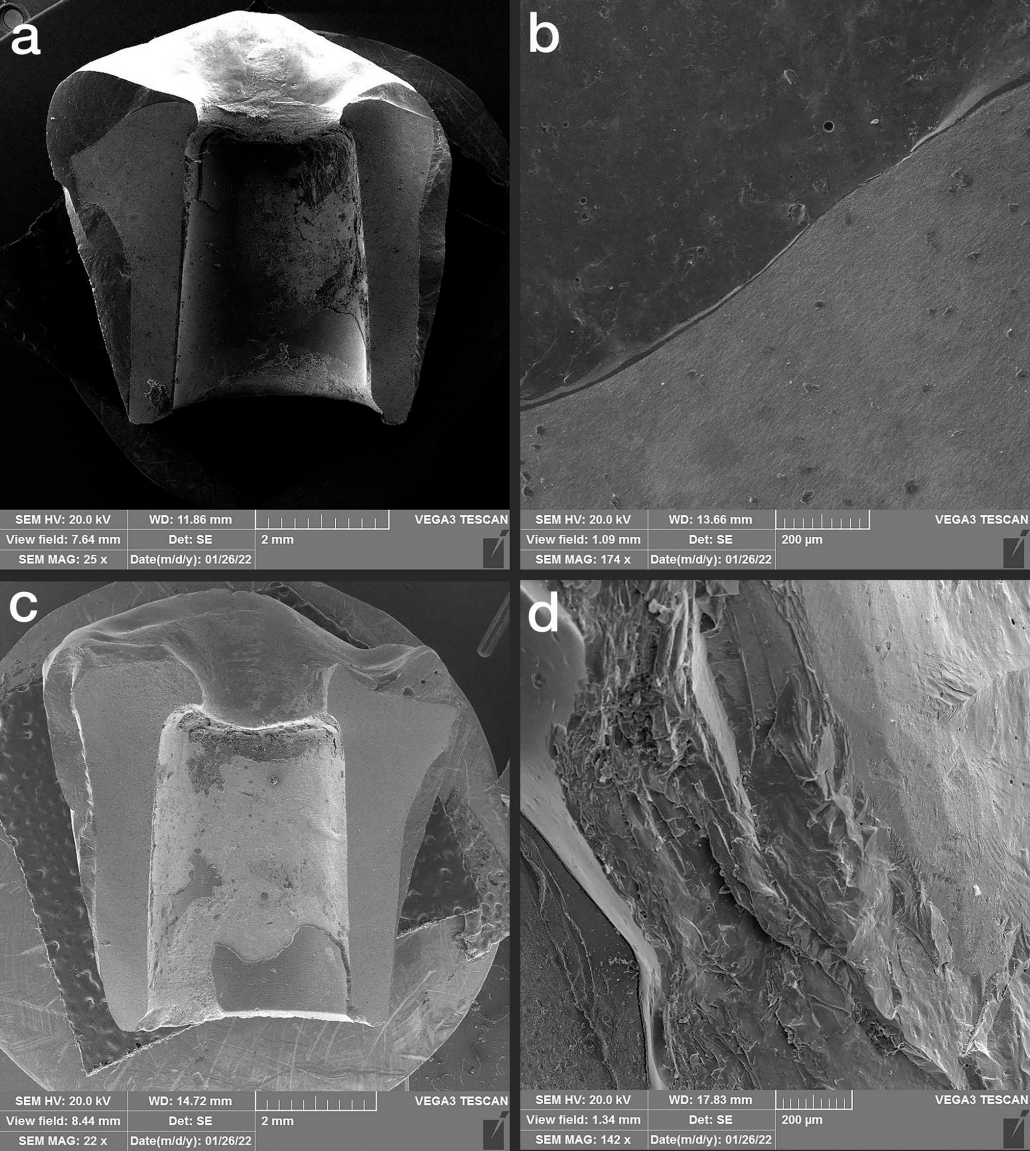
SEM images of porcelain-veneered zirconia specimen. a and b: thermocycled, c and d: non-thermocycled.

Half of the samples of each group (n = 7) were subjected to 10000 thermal cycles (5–55°C with a dwell time of 1 minute at each temperature), which equals one-year use of the restorations in the oral environment according to previous studies [19, 20]. During the artificial aging process, the specimens were inspected for any screw loosening. A vertical compressive load was applied on the specimens by a universal testing machine (ZwickRoell Z020, Germany) with a crosshead speed of 0.5 mm/min until failure or a significant drop in the load curve along with an evident crack sound. The fracture load for each specimen was recorded (**Fig 3**). The failure modes of the specimens were observed under a scanning electron microscope (SEM) (Vega3, TESCAN, Czech Republic) and classified as the porcelain fracture (fracture within the porcelain), interface fracture (fracture of the interface between the veneer and framework), and framework fracture (fracture within the framework) [1] (**Figs 4–6**).

### 2.3. Statistical analyses

The data were analyzed by using SPSS software (Version 16.0. Chicago, SPSS Inc., United States) The mean values and standard deviations were calculated for each group. Shapiro-Wilk test was used to evaluate the normal distribution, and Levene’s test was used to assess the equality of variances. Two-way ANOVA was used to evaluate the effect of thermocycling and type of restoration and the interaction effect of two factors on the fracture resistance of implant-supported restorations. One-way ANOVA was used to find differences between the restoration groups in each level of thermocycling, and Bonferroni’s post hoc test was used for pairwise comparison of the restoration groups. Independent samples t-test was used to compare the thermocycled and non-thermocycled subgroups in each restoration group (*α* = 0.05 in all tests).

## 3. Results

The mean (±standard deviation) of fracture load according to two levels of thermocycling and MZ, PVZ, and PFM restorations are summarized in **Table 1**. The results showed that the two-way ANOVA model was significant (*P* = 0.002 and F = 5.86, R2 = 0.947). Accordingly, the effect of restoration type (*P* = 0.005), thermocycling (*P* = 0.026), and the interaction effect of the two factors (*P*<0.001) were all statistically significant (**Table 2**).

**Table 1:** Mean and standard deviations and multiple comparisons of fractural load among the study groups (N).

Groups Subgroups	MZ	PVZ	PFM	P value *
Thermocycled	1143.7±187.2^A^	856.0±437.06^A^	983.4±233.6^A^	0.274
Non-thermocycled	1435.7±310.6^A^	1002.2±164.1^B^	1137.7±196.8^AB^	**0.008**
P value **	**0.055**	0.455	0.206	

* P values from one-way ANOVA

** P values from independent samples t-test

**Table 2:** Statistical results of the two-way ANOVA model.

Source	Type III sum of squares	df	Mean square	F	Sig.
Corrected model	1342025.524	3	447341.841	5.860	0.002
Intercept	50188374.857	1	50188374.857	657.476	0.000
Thermocycling	409664.381	1	409664.381	5.367	0.026
Group	932361.143	2	466180.571	6.107	0.005
Error	2900725.619	38	76334.885		
Total	54431126.000	42			
Corrected total	4242751.143	41			
R2 = 0.947					

**Fig 7:**
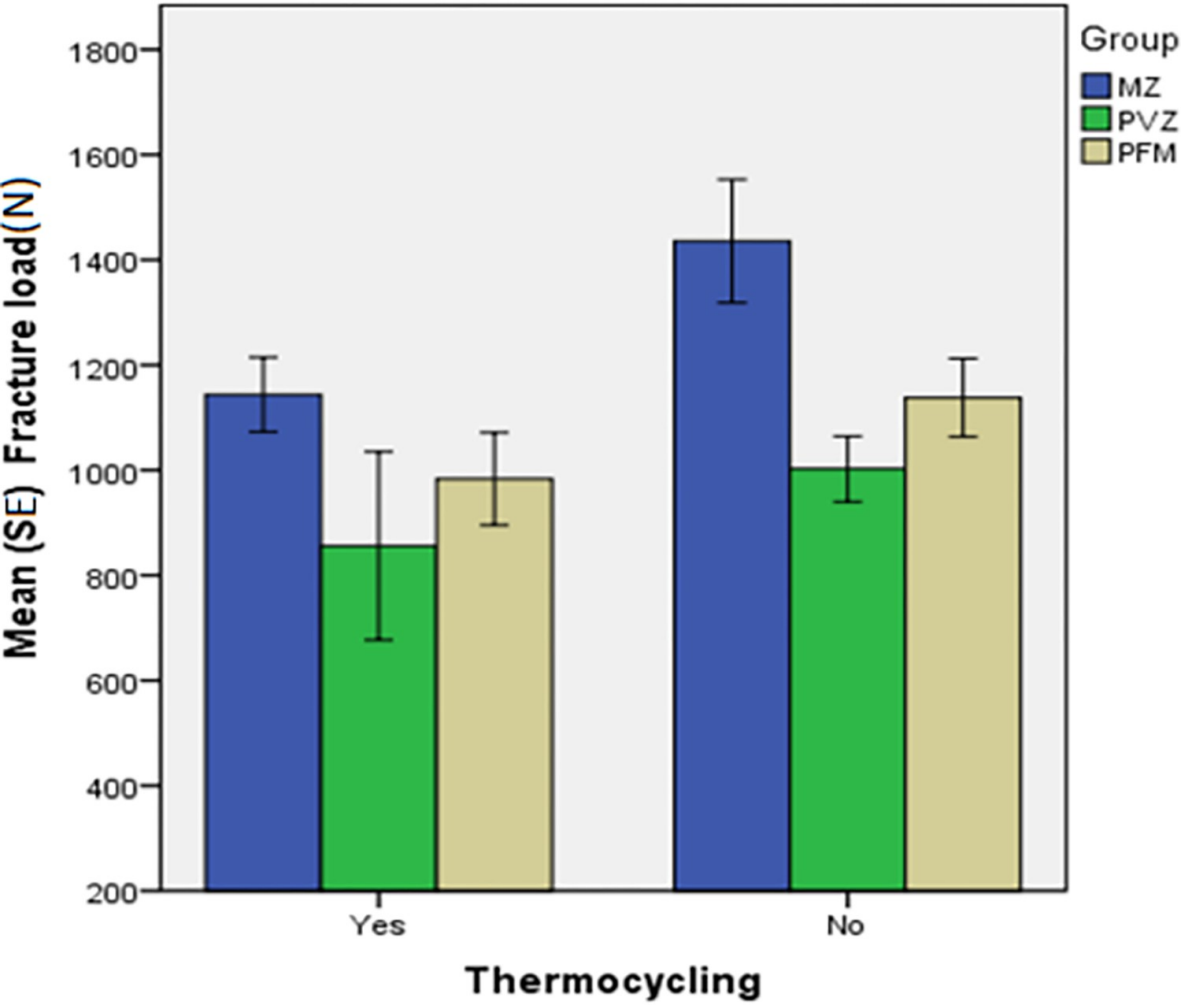
The mean and standard error (SE) of fracture load in the study groups.

**Fig 8:**
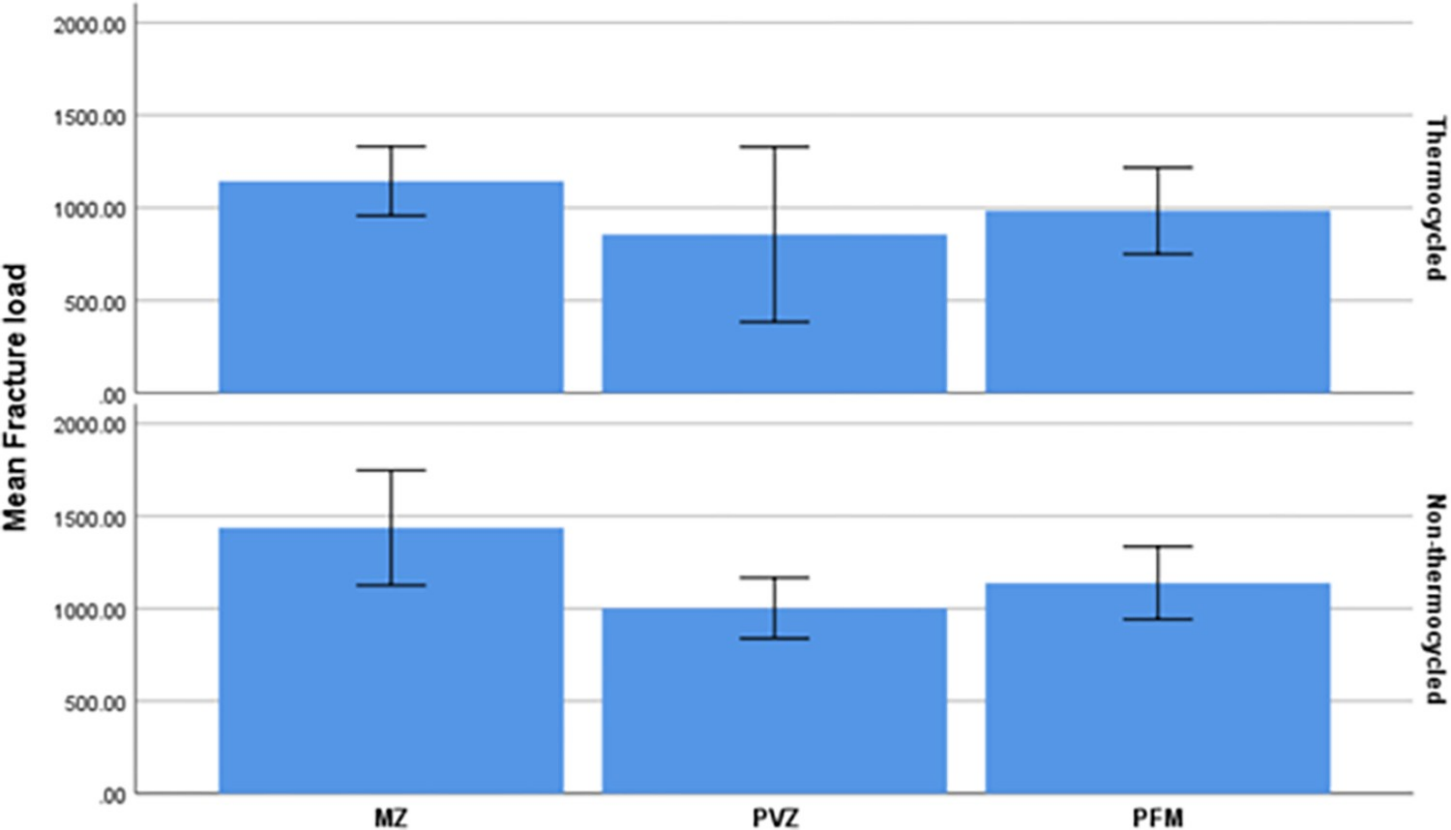
Pareto curve of MZ, PFM, and PVZ in thermocycled and non-thermocycled samples.

The result of one-way ANOVA revealed that the non-thermocycled subgroups had significantly different fracture resistances (*P* = 0.008). However, this difference was not statistically significant among the thermocycled subgroups (*P* = 0.274) (**Table 1**). Bonferroni’s post hoc test showed that the non-thermocycled MZ group was significantly more fracture-resistant (*P* = 0.008) than the non-thermocycled PVZ group. Independent samples t-test showed that thermocycling insignificantly decreased the fracture resistance in all the three MZ (*P* = 0.055), PVZ (*P* = 0.455), and PFM (*P* = 0.206) restoration groups (**Fig 7**). The Pareto curve of MZ, PFM, and PVZ in thermocycled and non-thermocycled samples was shown in **Fig 8**.

According to the SEM analysis, the failure mode, in the MZ group was full framework fracture along the screw access hole, which was the narrowest part of the restoration in all thermocycled and non-thermocycled specimens (100% framework fracture). In the PVZ group, 57% of specimens both thermocycled and non-thermocycled specimens showed fractures within the veneering porcelain. Moreover, 43% of specimens in the PVZ group showed framework fracture regardless of thermocycling. In the thermocycled PFM subgroup, most of the fractures (71%) were in the veneering porcelain; in non-thermocycling subgroups, 43% of failures were in the interface and 57% were in the veneering porcelain. (**Table 3**).

**Table 3:** Frequency of failure modes in the study groups (%).

Fracture mode Group		Veneering porcelain	Framework	Interface
Thermocycled	Monolithic zirconia	0	100	0
	Porcelain-veneered zirconia	57	43	0
	Porcelain-fused-to-metal	71	0	29
Non- thermocycled	Monolithic zirconia	0	100	0
	porcelain-veneered zirconia	57	43	0
	Porcelain-fused-to-metal	57	0	43%

## 4. Discussion

The two null hypotheses were rejected since both the type of restoration and thermocycling significantly affected the fracture resistance of screw-cement-retained restorations.

Before releasing material for clinical use, its performance and applicability should be tested. Although clinical studies are much more reliable for assessing the clinical success of materials like restoratives, they are sometimes replaced by in-vitro studies, which are quicker, reproducible, and allow using standardized test parameters. Moreover, their results are clinically more generalizable when the tests closely simulate the clinical conditions like the thermocycling in this study [21].

### 4.1. The effect of type of screw-cement retained restorations

The present findings showed that the MZ specimens were more fracture-resistant than the PVZ and PFM specimens before thermocycling. In agreement with the current results, Rosentritt et al. [22] and Augstin-Panadero et al. [23] detected that PVZ and PFM restorations had comparable fracture resistance. Likewise, Hussein et al. [11] assessed implant-supported restorations without thermocycling and noted that fracture resistance of restorations with screw access holes was not significantly different from those without screw access holes. Also, the MZ group had the highest fracture load both in groups with access holes and groups without access holes.

Zhang et al. [24] demonstrated that the structure of MZ ceramics could potentially optimize the restoration performance, owing to its material and geometric properties, as well as the elimination of the interface between layering materials and zirconia frameworks. This is a weak link in bilayered restorations; hence, its absence lowers the failure risk. Honda et al. [2] detected that the monolithic specimens had a significantly higher mean fracture resistance than bilayered restorations without thermal stress. Their findings support the results of the present study.

According to a study by Brizuela-Velasco et al. [25], the higher fracture resistance of the MZ specimens may be explained by the mechanical properties of zirconia like the excellent strength, hardness, and resistance to crack propagation, in addition to a narrow range of strength variation compared with porcelain. They found that the screw-retained monolithic high-translucency zirconia was more fracture-resistant than the high-translucency zirconia + feldspathic ceramic crowns. It was in agreement with the present results. Similarly, Elshiyab et al. [26] observed that monolayer hybrid-abutment crown structures were significantly more fracture-resistant than the bilayer counterparts when not thermocycled.

Lameira et al. [17] evaluated the effect of design and surface finishing on the fracture strength of yttria-tetragonal zirconia polycrystal crowns in monolithic and bilayer configurations after artificial aging. In contrast with the present study, they found that MZ crowns had higher fracture strength than the bilayer configuration. Taguchi et al. [27] evaluated the fracture resistance of single-tooth implant-supported zirconia-based indirect composite-layered molar restorations. They observed no significant difference in the fracture resistance between the PFM group and zirconia-based all-ceramic restorations. It contrasted the result of the present study, which demonstrated the monolithic zirconia to have significantly higher fracture resistance than the PFM group before thermocycling.

Another contrasting study was the one by Johansson et al. [16], who studied the fracture resistance of monolithic and bilayered zirconia and lithium disilicate glass-ceramic crowns after thermal and mechanical stress. Their results do not support the result of the present study because their MZ restorations had higher fracture resistance than bilayered restorations after thermal stress. Similarly, Honda et al. [1] concluded that the thermocycled MZ specimens were more fracture-resistant than the bilayered restorations since there was no interface between the veneer and zirconia framework, and also because the zirconia frameworks had fewer mechanical defects. It contrasted the results of the present study.

### 4.2. The effect of thermocycling

Thermocycling decreased the fracture strength in all the screw-cement retained restorations. Although this was not statistically significant, it can be considered clinically significant. These testing procedures have been discussed in previous studies as having a detrimental effect on the dental ceramics which resembles the clinical situation [28–30]. Cotes et al. [31] evaluated the effects of different aging methods on the degradation and flexural strength of yttria-stabilized tetragonal zirconia. Their results showed that mechanical and thermomechanical aging reduced the mechanical strength of zirconium dioxide ceramic. This is in line with the present results.

Elshiyab et al. [26] reported that non-aged monolayer zirconia crowns were significantly more fracture-resistant than both non-aged and aged bilayer zirconia. It was closely consistent with the findings of the current study; however, the monolithic zirconia was not significantly more fracture-resistant than the bilayered specimens after thermocycling. In the meantime, thermocycling did not statistically significantly affect the fracture strength of the control bilayered zirconia subgroups, which approves the result of the present study. They also found that thermal stress significantly reduced the fracture resistance of monolithic zirconia; which contrasted with what was found in the present study. They attributed this finding to the interaction of water with yttrium and the produced yttrium hydroxide, which eventually leads to yttrium deficiency, triggering the transformation of zirconia from the stable tetragonal phase to the less stable and weaker monoclinic phase [26, 32].

Another study by Elshiyab et al. [33] concluded that hydrothermal stress reduced the fracture resistance of MZ crowns. However, unlike the current study, the difference was statistically significant. Reduced fracture resistance of the MZ crowns was due to the aging of zirconia, also known as “low-temperature degradation” of zirconia crystal, in which tetragonal crystal phases turn into the less stable monoclinic phase. Temperature plays a key role in the aging process of zirconia [31].

### 4.3. Fracture modes

Porcelain fracture happened in both types of layered restorations (PVZ and PFM) with or without thermocycling. This is because the screw access hole weakens the porcelain around the opening and at the cusp tip and disrupts its integrity [34]. This is in agreement with Karl et al.’s findings [35], which concluded that screw-retained restorations had more porcelain chipping fractures in comparison with the cement-retained restorations. It is probably because the screw access hole may be a weak point of the ceramic veneer of implant-supported restorations. Saito et al. [36] asserted that strong discrepancies in the Coefficient of thermal expansion between the veneering porcelain and zirconia significantly affected their bond strength. This also may be the reason for interface fracture in the PVZ group.

In the PVZ group, the fracture was most frequent within the veneering porcelain, followed by the framework; but, never in the interface between the zirconia framework and the veneering porcelain. In the PFM group, fracture occurred both within the veneering porcelain and in the interface between metal coping and the veneering porcelain. This was in line with Mallmann et al.’s study [13], which reported that, unlike the zirconia-based restorations, porcelain fracture in restorations with metal frameworks mostly reached the framework. This is because of the adequate interfacial bond between the zirconia and porcelain.

Lameira et al. [17] evaluated the fracture modes of polished MZ crowns, glazed MZ crowns, and bilayer crowns. According to their findings, a fracture within the veneering layer happened most commonly in restorations with the powder build-up technique, which is the technique used in the present study to fabricate the PVZ group, rather than in the sintering or pressed veneering technique. They concluded that the powder build-up technique is a technique-sensitive process and requires many firing cycles; thus, it may increase the addition of impurities and porosities, which maximizes the risk of crack propagation.

Johansson et al. [16] also observed the fracture modes for veneered high translucent zirconia (Y-TZP) crowns and veneered lithium disilicate crowns. They found that veneered restorations exhibited both total and cohesive fractures through the porcelain-veneer only but no adhesive fractures. This is against the findings of the present study in which also interface fractures were seen in the PVZ group.

All of the investigated types of screw-cement retained implant-supported zirconia-based restorations appear to be resistant to masticatory forces of the premolar region which is about 220–450 N [37–39].

Among the limitations of this in-vitro study was the use of only single crowns instead of multi-unit implant-supported screw-cement retained restorations. Also, only two types of implant-supported zirconia-based restorations were studied in this experiment; further studies are suggested to assess restorations like indirect veneered zirconia crowns.

## 5. Conclusions

Within the limitations of this study, the following conclusions can be drawn:

1. Without thermocycling, the fracture resistance of the MZ group was significantly higher than the PVZ and PFM groups; while after thermocycling, there was no statistically significant difference between the fracture resistance of different restoration groups.
2. Although thermocycling could decrease the fracture resistance of each restoration type, it was not statistically significant; however, it can be clinically important.
3. All of the fractures (100%) in the MZ group were in the framework along the screw access hole both with and without thermocycling. In the PVZ group, 57% were veneering porcelain fractures and 43% were interface fractures for both thermocycling subgroups. In the PFM group, 57% of failures were the veneering fracture in non-thermocycled specimens and it increased to 71% in thermocycled specimens.

## Supporting information

S1 Data (XLSX)
